# Impact of Breath Holding on Spleen Stiffness Measured by 100 Hz Vibration Controlled Transient Elastography

**DOI:** 10.1111/liv.70714

**Published:** 2026-06-13

**Authors:** Sebastiana Atzori, Pierluigi Meloni, Marco Arru, Gianpaolo Vidili

**Affiliations:** ^1^ Department of Internal Medicine Azienda Ospedaliero Universitaria di Sassari Sassari Italy; ^2^ Department of Medicine, Surgery and Pharmacy University of Sassari Sassari Italy; ^3^ Day Hospital of the Medical Area, Department of Internal Medicine Azienda Ospedaliero Universitaria di Sassari Sassari Italy

**Keywords:** non‐invasive ultrasound methods, portal hypertension, spleen stiffness, transient elastography

## Abstract

**Backgrounds and Aims:**

Ultrasound‐based spleen stiffness measurement (SSM) is a valid non‐invasive tool to assess portal hypertension (PH) in chronic liver disease. Whereas the role of the respiratory phase during liver stiffness measurements is established, no study has specifically addressed how respiration influences SSM by transient elastography.

**Aims:**

To evaluate the influence of respiration on SSM assessed with FibroScan 630 (Echosens, Paris, France).

**Methods:**

Eighty‐three patients with chronic liver disease of different aetiologies underwent SSM using vibration‐controlled transient elastography (VCTE).

**Results:**

SSM acquired during a normal respiratory cycle showed better diagnostic accuracy than measurements obtained during breath‐hold after deep inspiration (AUROC 0.835 [95% CI 0.743–0.928] vs. 0.798 [95% CI 0.697–0.899]).

**Conclusion:**

SSM by VCTE should be performed during quiet breathing, as it showed good diagnostic accuracy for predicting the presence of oesophageal varices (OV) in patients with chronic liver disease.

## Key Points

Spleen stiffness measurement by vibration‐controlled transient elastography showed higher diagnostic accuracy for predicting oesophageal varices during quiet breathing than during breath‐hold after deep inspiration, supporting the use of normal respiration during assessment.

## Lay Summary

This study shows that measuring spleen stiffness—a non‐invasive method to assess stiffness in the spleen—works best when patients breathe normally, rather than holding their breath. Measurements taken during quiet breathing were more accurate in detecting patients with oesophageal varices. This simple adjustment could make the test more accurate in everyday clinical practice

## 1 Introduction

Portal hypertension (PH) is a major driver in the progression from compensated to decompensated cirrhosis, characterised by clinical complications such as ascites, gastroesophageal bleeding, spontaneous bacterial peritonitis, hepatorenal syndrome, and hepatic encephalopathy [1]. The hepatic venous pressure gradient (HVPG) is considered normal up to 5 mmHg; subclinical PH is defined by an HVPG of 6–9 mmHg, while an HVPG of 10 mmHg or more corresponds to clinically significant portal hypertension (CSPH) [2]. PH may occur before a formal anatomical diagnosis of cirrhosis, leading to the proposed entity of compensated advanced chronic liver disease (cACLD) [3]. This entity includes patients with cirrhosis and those with advanced liver fibrosis and PH.

Oesophageal varices are dilated submucosal veins at the gastroesophageal junction that develop as a direct consequence of sustained portal hypertension. They represent one of the most severe complications of chronic liver disease, with variceal haemorrhage accounting for a major cause of mortality in cirrhotic patients [4].

HVPG and endoscopy are current gold‐standard techniques to assess portal hypertension. However, they are invasive, not always well‐tolerated, carry a risk of complications, and require specialist training and equipment to be carried out.

Spleen enlargement is a well‐known consequence of liver cirrhosis. It is due to tissue hyperplasia and to portal congestion and hypertension and splenic fibrosis [5]. On this basis, more recently, spleen stiffness measurements (SSM) using non‐invasive ultrasound‐based methods have emerged as an important alternative method to detect portal hypertension [6]. Several studies, most of which were performed with vibration‐controlled transient elastography, have shown that, in patients with portal hypertension, spleen stiffness is more reliable than liver stiffness [7, 8, 9, 10]. The diagnostic performance obtained from these studies was reasonably good, and specificity and sensitivity were greater than 70% in most of the cases. However, the range of cutoff values is wide, ranging from 47.6 to 56.3 kPa for CSPH and from 40.8 to 65 kPa for detecting any OVs. For large varices, cutoffs are narrow, ranging from 54 to 54.5 kPa, but only a few studies have been published [11].

Results regarding the diagnostic accuracy of SSM obtained using VCTE in predicting CSPH are disputable and affected by limitations such as the high rate of failure in small spleens and a maximal detectable value of 75 kPa [12]. To overcome these limitations, a novel spleen‐dedicated examination based on VCTE has been developed. The Fibroscan 630 Expert device is equipped with a B‐mode ultrasound probe to help localise the spleen and has spleen‐dedicated VCTE settings for the M probe with a fixed frequency of 100 Hz [13].

Some recent studies demonstrated a good correlation between portal hypertension and spleen stiffness measured by the novel Fibroscan 630 Expert device [11, 14].

Although some relevant recent multicentre studies demonstrated a good accuracy of spleen stiffness to detect portal hypertension, the spleen stiffness measurement is not standardised and one of the main issues in this sense is to establish in which phase of respiration it should be performed. To our knowledge there are no studies concerning the best respiratory phase of the spleen stiffness measurements compared to those for liver stiffness.

In a clinical setting, spleen stiffness measurements cannot always be performed in a standardised manner during a normal respiratory phase, and it could be difficult to reach by the transducer. Thus, it is important to exclude potential confounding factors that can reduce the accuracy of spleen stiffness measurements.

The impact of the respiratory phase on spleen stiffness variability has not yet been investigated.

The aim of this study was to investigate the influence of respiration on spleen stiffness measurements assessed with Fibroscan 630 (Echosens, Paris, France) in patients with cirrhosis and PH.

## 2 Materials and Methods

This is a prospective observational study approved by the Local Research Ethics Committee in accordance with the Helsinki Declaration of 1975 [15] (6th revision 2008) and then approved by the Ethics Committee of AOU of Cagliari (protocol code PG/8821).

Written informed consent was obtained from all patients. Eighty‐three patients with a diagnosis of liver cirrhosis, scheduled for a follow‐up routine appointment at the Day Hospital Unit of the Medical Area, Department of Internal Medicine, Azienda Ospedaliero Universitaria (AOU) Sassari, were enrolled over a one‐month period from the 1st of September 2023 to the 30th of September 2023. VCTE was performed to simultaneously assess spleen stiffness in two patient groups. The patients were divided into the following groups: patients with oesophageal varices (OGD) (group 1) and patients with no oesophageal varices (group 2).

Groups were matched for age. Inclusion criteria included the ability and willingness to provide written informed consent, age between 18 and 75, presence of cirrhosis, and willingness to consent to medical note and diagnostic test review by the clinical research team. Exclusion criteria included pregnancy, absence of liver disease pathology, and trans jugular portosystemic shunt (TIPS) insertion.

All patients were studied in the morning after an overnight fast. Participants were placed supine with arms abducted away from the ultrasound probes.

Cirrhosis was diagnosed based on histologic examination or combined clinical, laboratory, and radiologic findings or signs of decompensation [16]. Parameters determining the presence of PH, such as oesophagogastro duodenoscopy (OGD) findings, were recorded. OGD for varices screening was part of routine clinical practice, with only endoscopies performed within 6 months from the elastographic assessment considered in the analysis.

### 2.1 Transient Elastography

VCTE was performed using the Fibroscan 630 (Echosens, Paris, France) with a dedicated M 100 Hz probe for SSM. An experienced operator, who had conducted more than 1000 liver procedures with Fibroscan and undergone a certified training session specifically for SSM with Fibroscan 630 with an Echosens consultant, performed all examinations. The spleen stiffness measurements were performed after the detection of the spleen with the dedicated ultrasound probe placed in the left intercostal space with the left arm extended above the head.

Clinical and biological parameters including body mass index (BMI), aspartate aminotransferase (AST), alanine aminotransferase (ALT), γ‐glutamyl transpeptidase (GGT), alkaline phosphatase (ALP), platelet count, prothrombin time, albumin, bilirubin and international normalised ratio (INR) were obtained for all patients at time of recruitment. AST to Platelet Ratio Index (APRI) score was calculated as: AST (IU/L)/PLT (×10^9^/L).

### 2.2 SSM According to Respiratory Phase

To assess variability during the respiratory phase measurements were taken as following:

1. Ten spleen stiffness measurements were taken during a normal respiratory cycle.
2. Ten spleen stiffness measurements were taken on a breath hold for few seconds.

For each set of measurements median value was calculated.

### 2.3 Statistical Analyses

Quantitative variables were expressed as mean ± SD or median (IQR), and qualitative variables as absolute and relative frequencies. Baseline demographic, laboratory and biopsy characteristics of the patients are summarised using descriptive statistics.

The distribution of the numerical variables was tested by Shapiro–Wilks test. The Central Limit Theorem was used to establish that normalised sum of independent variables tends toward a normal distribution. Bonferroni corrections were applied.

Correlations between variables were examined using Pearson's correlation coefficient, and *p* values determined using ANOVA. Diagnostic accuracy was assessed using ROC curves, with Youden's index determining cut‐off values [17, 18]. The ROC curves were compared using the nonparametric approach of DeLong test. Inter‐operator variability was assessed using a one‐way random interclass correlation coefficient (ICC) model. *p* Values < 0.05 were considered statistically significant.

All statistical analyses were performed using SPSS version 29.0.2.0.

## 3 Results

**FIGURE 1:**
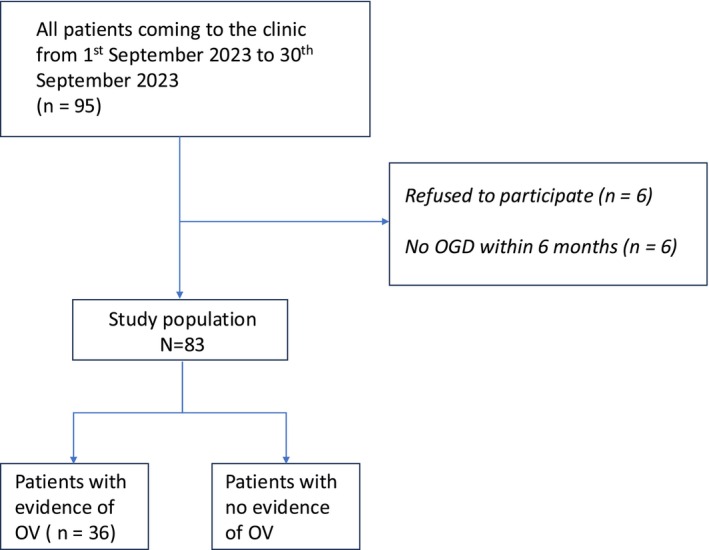
Flow chart of the study protocol. Abbreviations: OGD, esophagogastroduodenoscopy; OV, oesophageal varices.

Ninety‐five patients were recruited. Twelve patients were excluded as summarised in Figure 1. A total of 83 patients (mean age 62.6 years, 62.0% male) were included: 36 with EV (mean age 62.09) and 47 without OV (mean age 60.71). Among the patients with oesophageal varices, two patients had as well gastric varices. No patients with portal vein thrombosis or splanchnic vein thrombosis. Baseline clinical and biochemical characteristics are summarised in Table 1.

**TABLE 1:** Characteristics of the study cohort.

	Group 1 (*n* = 36)	Group 2 (*n* = 47)
*Gender*		
Female	7	14
Male	29	33
Age, years old	63.13	62.13
BMI, kg/m^2^	26.15	26.54
*Laboratory finding*		
Haemoglobin (g)	11.89	13.56
Platelet, 10^3^/mm^3^	97.22	225.26
AST (IU/L)	36.68	27.42
ALT (IU/L)	26.04	27.09
Bilirubin (mg/dL)	2.24	0.51
GGT/Platelet	0.73	0.27
Albumin (g/L)	3.20	3.78
INR	1.13	1.11
APRI	1.07	0.35
GGT (IU/L)	81.17	68.78
*Spleen stiffness measurements according to respiratory phase*		
VCTE SSM, median (kPa)	68	24 p < 0.001***
VCTE BH‐SSM, median (kPa)	47	21 p < 0.001***
*Aetiology*		
Chronic Viral Hepatitis C	22	19
Chronic Viral Hepatitis B		2
AIH		3
Alcohol related liver disease	12	12
PBC		3
MASH	1	3
Cryptogenic		3
Wilson		1
Sickle Cell Anaemia		1
HIV	1	
Hepato‐cellular carcinoma HCC	9	2
Ascites	13	1
Past medical history of ascites		1

*Note:* Baseline characteristics of the patients. Data are mean; Group 1: Population with evidence of oesophageal varices; Group 2: Population with no evidence of oesophageal varices. Spleen stiffness measurements by vibration controlled transient elastography during a normal respiratory cycle (SSM) and during a breath hold (BH‐SSM). Statistical significance *p* < 0.001***.

Abbreviations: AIH, autoimmune hepatitis; ALD, alcohol liver disease; ALT, alanine aminotransferase; AST, aspartate aminotransferase; BH‐SSM, breath‐hold spleen stiffness measurements; BMI, body max index; GGT, gamma‐glutamyl transpeptidase; HCC, hepato‐cellular carcinoma; HIV, human immunodeficiency virus; kPa, kilo Pascal; MASH, metabolic dysfunction‐associated steatohepatitis; PBC, primary biliary cholangitis; SSM, spleen stiffness measurements.

### 3.1 Effects of Breath Phase on Spleen Stiffness and Accuracy

**FIGURE 2:**
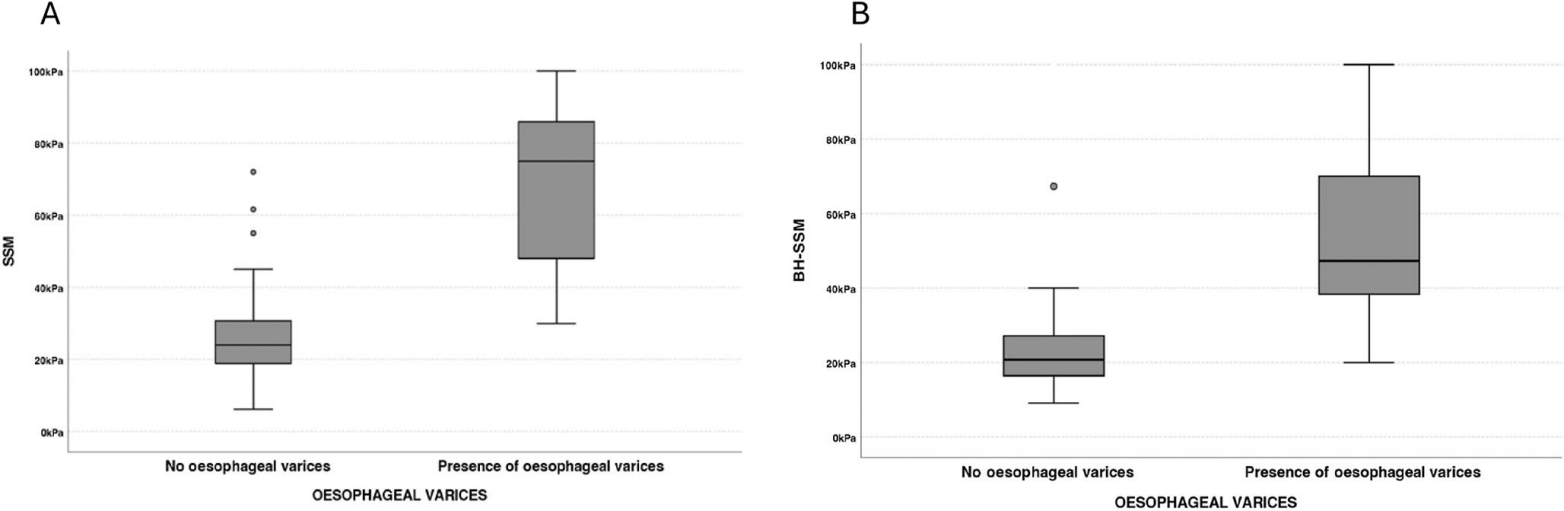
Boxplots of the spleen stiffness values of SSM during a normal respiratory phase (SSM) (A on the left size) and during a breath hold (BH‐SSM) (B on the right size) in both groups, respectively without and with oesophageal varices. The boxes represent the interquartile range, and the thick lines within boxes, the median values measured using SSM and BH‐SSM. The error bars indicate the smallest and largest values within 1.5 box lengths of the 25th and 75th percentiles, respectively. The dots are outliers representing very large values that deviate significantly. BH‐SSM, breath‐hold spleen stiffness measurements; SSM, spleen stiffness measurements.

SSM both during a normal respiratory phase and during a breath holding were significantly higher in patients with oesophageal varices (Figure 2).

Median values of SSM taken during a normal respiratory cycle in patients with oesophageal varices were significantly higher compared to the measurements taken with a breath‐holding (68.6 and 47 kPa respectively). In patients with no oesophageal varices, there was no statistically significative difference in the SSM taken during a normal breath and during a breath holding (24 and 21 kPa respectively).

**FIGURE 3:**
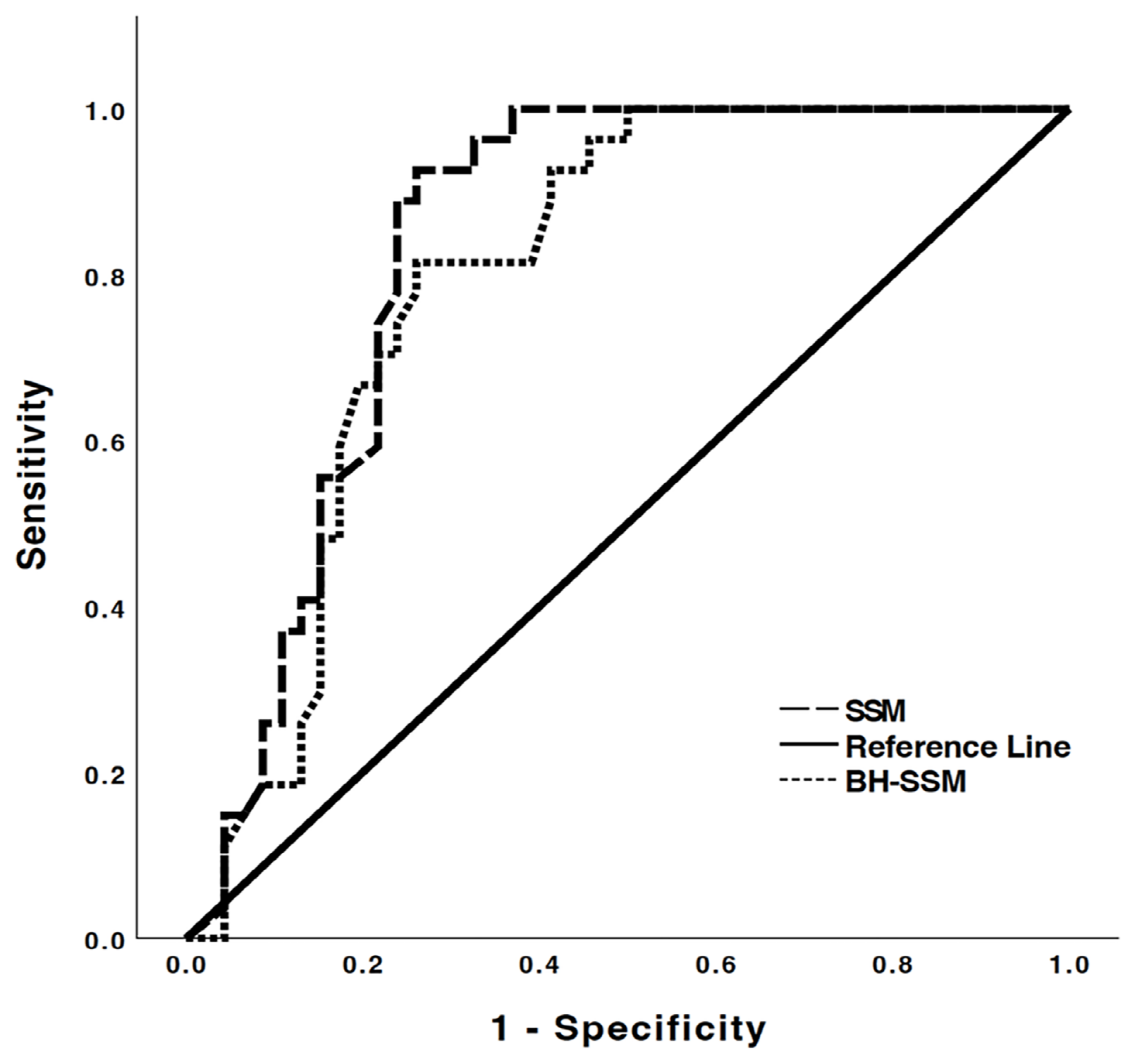
Roc curves of spleen stiffness during a normal respiratory cycle (SSM) and during a breath hold (BH‐SSM) diagnostic performance. BH‐SSM, breath‐hold spleen stiffness measurements; SSM, spleen stiffness measurements.

Spleen stiffness measurements during a normal respiratory cycle showed a better accuracy compared to the SSM taken during a breath hold after a deep inspiration (AUROC 0.835 (95% CI 0.743–0.928) 0.798 (95% CI 0.697–0.899) respectively) (Table 2) (Figure 3). The pairwise comparisons of the SSM values showed that there was significant difference between SSM during a normal respiratory cycle and SSM during a breath holding (*p* 0.058*).

**TABLE 2:** Performance of spleen stiffness measurements by vibration controlled transient elastography of Spleen stiffness measurements by vibration controlled transient elastography during a normal cycle (SSM) and during a breath hold in inspiration (BH‐SSM) in predicting the presence of oesophageal varices.

TEST	AUROC (95% CI)	Cut‐off kPa (OVs 0/1)	SE	SP	PPV	NPV	*p*
VCTE SSM	0.836 (0.743–0.928)	37.6	0.889	0.739	0.895	0.861	< 0.01**
VCTE BH‐SSM	0.798 (0.697–0.899)	31.3	0.814	0.733	0.884	0.824	< 0.01**

*Note:* Receiver operating characteristic curves for the prediction of oesophageal varices. Data are expressed with 95% confidence interval. Statistical significance *p* < 0.01**.

Abbreviations: AUROC, area under the receiver operating characteristic curve; BH‐SSM, breath‐hold spleen stiffness measurements; kPa, kilo Pascal; NPV, negative predictive value; PPV, positive predictive value; SE, sensitivity; SP, specificity; SSM, spleen stiffness measurements; VCTE, vibration controlled transient elastography. OV, oesophageal varices.

**FIGURE 4:**
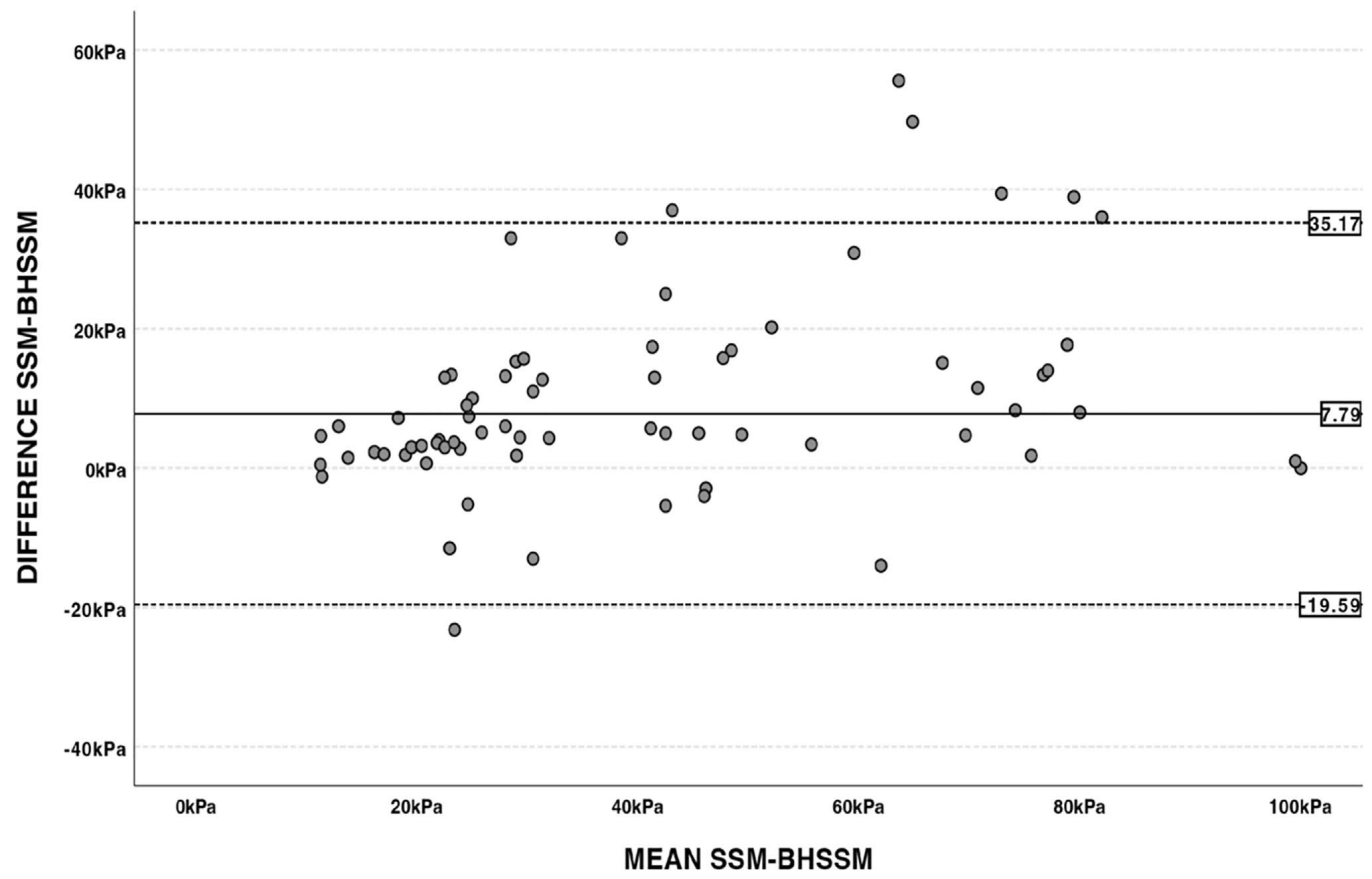
Bland–Altman plot of differences between spleen stiffness measurements during a normal respiratory cycle (SSM) and during a breath hold (BH‐SSM) and the mean of the two. The black line indicates the mean difference between the two methods, while the dashed lines delimit the 95% confidence interval of the differences.

The interclass correlation coefficient (ICC) showed SSM during a normal respiratory cycle/SSM during a breath holding ICC 0.633 (95% CI 0.601–0.722). Bland–Altman analysis (Figure 4) with one‐way *t*‐test indicates that the mean difference values between the two sets of measurements was 7.79 with statistically significant systematic bias (*p* 0.001***).

## 4 Discussion

Liver stiffness measurements (LSM) have been used since many years to assess severity and prognosis in patients with liver diseases [19] and nowadays Spleen stiffness evaluation using transient elastography is increasingly being used for detecting portal hypertension as stated in the Baveno VII guidelines [3].

A recent multicentre study showed that SSM by Transient Elastography < 40 kPa combined with bilirubin < 1 mg/dL defined patients with portal hypertension with a probability of high‐risk varices < 5% in whom screening endoscopy could be spared [14].

Previous studies showed similar diagnostic accuracies of SSM and LSM in predicting EV [20, 21, 22]. Another recent study showed that liver stiffness and Doppler study combined had a good accuracy to predict the presence and the severity of OV [23].

Spleen area, spleen stiffness and platelet count may be useful markers to assess the presence of portal hypertension in patients of various etiologies.

Despite the increasing numbers of evidence and studies [24, 25] on spleen stiffness measurements by Fibroscan 630, cut off values still show a high variability among the different studies due probably different aetiologies but also a lack of standardisation of the procedure.

In particular, going deeply in the methodology of the studies, it is not well declared the breathing phase by which the spleen stiffness measurements have been taken [26, 27].

It is very important that the determination of spleen stiffness is standardised as much as possible to ensure reliable measurements. In this context, we investigated the effect of breathing phases on spleen stiffness.

The main finding of our study was that spleen stiffness measurements by Fibroscan 630 showed a significant higher stiffness values during a normal respiratory cycle compared to the measurements taken during a breath hold in inspiration. Spleen stiffness measurements during a normal respiratory cycle showed a better accuracy compared to the SSM taken during a breath hold after a deep inspiration (AUROC 0.836 (95% CI 0.743–0.928) 0.798 (95% CI 0.697–0.899) respectively).

The reason for observing a lower spleen stiffness value during inspiration followed by breath‐hold is likely due to the potential decongestion of the spleen. During inspiration, the decrease in intrathoracic pressure enhances the flow from abdominal veins toward the thorax. Specifically, blood flow from the liver to the heart increases, and simultaneously, flow from the spleen to the portal vein also rises. This phenomenon has not been previously studied, and these findings suggest that the effects of respiration should be considered when evaluating spleen stiffness using Fibroscan 630.

A previous study showed that an increase in pressure and a decrease in blood flow volume occurs in the portal venous system during inspiration and the reverse conditions occur during expiration [28].

Although several factors may contribute to these observations, the main explanation is believed to be that inspiration temporarily interrupts splanchnic venous outflow. This occurs because contraction of the diaphragm compresses the liver parenchyma, leading to mechanical narrowing or collapse of the intrahepatic vessels and a consequent rise in intrahepatic vascular resistance. In contrast, during expiration, portal venous blood flow tends to increase [29].

In cases of liver cirrhosis, splenic venous return is significantly affected due to portal hypertension, a common condition in cirrhosis. Portal hypertension causes a retrograde increase in pressure within the splenic vein, leading to venous congestion. Consequently, the splenic vein must “work” against higher pressure to drain blood toward the portal vein, resulting in a slower, more challenging venous return.

Due to high resistance in the portal system, blood from the splenic vein cannot efficiently drain through the liver, resulting in ineffective splenic venous return. Deep breathing could improve the decongestion of the spleen flow. Measuring spleen stiffness during breath‐hold inspiration should be avoided, as it may obscure actual spleen congestion, which contributes to the organ's stiffness and indicates the presence of portal hypertension.

Our study showed statistically significative reduction of accuracy of spleen stiffness during the breath hold phase. It therefore appears important to standardise the procedure for future studies and for clinical practice to obtain more comparable results between individual patients and to allow a more precise monitoring of patients over time.

Our study showed the importance of an optimised protocol for breathing phase for spleen stiffness measurements using VCTE Fibroscan 630 avoiding deep inspiration prior to scanning.

Our study is limited by a relatively small number of included participants. Moreover, patients were not excluded based on pharmacological treatment for PH, as non‐selective beta blockers and variceal banding were unlikely to affect splenic measurements.

We did not perform HVPG measurements, the gold standard for evaluating PH presence, but clinically significant portal hypertension (CSPH) was confirmed via OGD.

## 5 Conclusions

Median values of SSM taken during a normal respiratory cycle in patients with oesophageal varices were significantly higher compared to the measurements taken with a breath‐holding (68.6 and 47 kPa respectively). SSM showed a good diagnostic accuracy to predict the presence of OV in patients with chronic liver disease.

## Author Contributions

**Sebastiana Atzori:** conceptualization, methodology, software, validation, formal analysis, writing – original draft preparation, writing – review and editing, visualization. **Pierluigi Meloni:** investigation. **Marco Arru:** validation, resources, data curation, writing – review and editing. **Gianpaolo Vidili:** conceptualization, methodology, software, validation, writing – review and editing, supervision, project administration, funding acquisition.

## Funding

This research and the relative APC were funded by Fondazione di Sardegna, grant number FDS2019VIDILI.

## Ethics Statement

The study was conducted in accordance with the Declaration of Helsinki and approved by the Ethics Committee of AOU of Cagliari (protocol code PG/28821).

## Consent

Informed consent was obtained from all subjects involved in the study.

## Conflicts of Interest

The authors declare no conflicts of interest.

## Data Availability

The data that support the findings of this study are available on request from the corresponding author. The data are not publicly available due to privacy or ethical restrictions.
